# Computational modeling and optimized machine learning for multidrug solubility prediction in supercritical carbon dioxide

**DOI:** 10.3389/fmed.2026.1985801

**Published:** 2026-10-16

**Authors:** Nidal H. Abu-Hamdeh, Hasan H. Hijji, Khalid H. Almitani

**Affiliations:** 1 Center of Research Excellence in Renewable Energy and Power Systems/Energy Efficiency Group, Department of Mechanical Engineering, Faculty of Engineering, King Abdulaziz University, Jeddah, Saudi Arabia; 2 Mechanical Engineering Department, College of Engineering and Architecture, Umm Al-Qura University, Makkah, Saudi Arabia; 3 Department of Mechanical Engineering, Faculty of Engineering, King Abdulaziz University, Jeddah, Saudi Arabia

**Keywords:** CatBoost, computational modeling, drug solubility, hyperparameter optimization, supercritical carbon dioxide, supercritical fluid processing, XGBoost

## Abstract

Reliable prediction of pharmaceutical solubility in supercritical carbon dioxide (scCO_2_) is complicated by strong compound-specific behavior and response values. This study investigated whether sparse measurements from a newly introduced drug can calibrate transferable machine-learning predictions for computational estimation of solubility in wide range of parameters. A curated workbook containing 1,618 observations for 60 drugs was modeled using molecular weight, melting point, temperature, and pressure. Solubility was transformed as log₁₀(S), and XGBoost, CatBoost, and multilayer perceptron regression were each tuned using tree-structured Parzen estimation, differential evolution, and particle swarm optimization. Evaluation used five-fold nested group cross-validation, with complete drugs held out from training and tuning. For each test drug, predictions were assessed before calibration and after 1, 3, or 5 randomly selected measurements, repeated ten times. Within the present evaluation framework, the lowest-log-MAE zero-shot configuration was XGBoost optimized by tree-structured Parzen estimation (log-MAE = 0.584; R² = −0.087; RMSE = 1.713 g/L), showing that the four descriptors were insufficient for reliable extrapolation to unseen compounds. Sparse calibration substantially improved transfer: the lowest-log-MAE one-shot result was obtained by CatBoost–particle swarm optimization (log-MAE = 0.418; R² = 0.166), whereas XGBoost–tree-structured Parzen estimation had the lowest log-MAE at three shots (log-MAE = 0.298; R² = 0.317) and at five shots (log-MAE = 0.251; R² = 0.452; RMSE = 1.223 g/L). Median absolute relative deviation decreased from 82.4% to 42.9% between zero and five shots. These results establish sparse, leakage-controlled calibration as a more defensible use case than unconditional prediction for unseen drugs, while identifying the need for richer molecular descriptors and external prospective validation. In practical terms, this few-shot strategy can reduce the experimental burden for a newly introduced drug by using a small screening set to calibrate a global model before mapping the remaining pressure-temperature domain.

## 1 Introduction

Drug solubility is a central determinant of formulation feasibility, bioavailability, particle engineering, and the selection of manufacturing conditions. Supercritical carbon dioxide has attracted sustained pharmaceutical interest because it is nonflammable, inexpensive, readily removed by depressurization, and compatible with several micronization and particle-formation processes (1–4). The process has been considered as green method which can be used for production of nanomedicines with enhanced solubility in aqueous solutions because the smaller size of particles would dissolve more in the solvent owing to the higher free energy. Its solvent power is nevertheless highly sensitive to pressure, temperature, density, solute volatility, and specific solute–solvent interactions. Experimental solubility measurements therefore remain indispensable but require specialized high-pressure equipment, careful equilibration, and repeated analyses over the intended process window (2, 3). As such, some recent works have recommended integration of experimental data to computational modeling to further develop supercritical processing for nanomedicines production.

Beyond serving as a tunable solvent, supercritical CO_2_ (scCO_2_) is important to pharmaceutical manufacturing because depressurization can support drug particle formation and micronization with little residual organic solvent, while supercritical-fluid routes can be integrated with formulation and encapsulation strategies used to produce micro- and nanoscale drug products (2–4). These attributes make scCO_2_ particularly relevant when particle size, morphology, dissolution behavior, and solvent exposure are critical to formulation performance and nanomedicine development.

Thermodynamic descriptions of pharmaceutical solubility based on equations of state, density correlations, and semi-empirical models can represent individual solutes when suitable critical properties and interaction parameters are available (3–6). Their physical interpretability is valuable, but compound-specific fitting and uncertain sublimation or critical-property estimates can limit transferability. Machine learning (ML) provides a complementary route by learning nonlinear associations from accumulated measurements. Recent studies have applied support-vector, ensemble-tree, boosting, and neural models to drug solubility in scCO_2_, including single-drug and multidrug datasets (7–13). Contemporary multidrug studies have reported strong aggregate accuracy when observations from the same compounds occur in both training and test subsets (11–13). Such random-row validation measures interpolation across known compounds, however, and should not be interpreted automatically as performance for a drug absent from model development (14–16). The key research gap is therefore not simply whether ML can fit accumulated scCO_2_ solubility data, but whether a model can transfer to a chemically unseen drug without benefiting from measurements of that same compound during development. ML can address this gap by learning shared nonlinear pressure-temperature-solubility patterns across compounds, provided evaluation prevents compound leakage and explicitly tests how much target-drug information is needed for useful calibration.

The distinction is especially important in pharmaceutical datasets, where repeated pressure–temperature measurements from one compound share molecular weight, melting point, and unmeasured chemical characteristics. Randomly splitting these rows can allow compound identity to be partially reconstructed and can produce optimistic estimates. Grouped validation, in which all observations for a drug remain in one fold, provides a stricter estimate of chemical transfer (14–17). Few-shot learning offers a pragmatic intermediate scenario: a global model supplies an initial response surface, and a small number of measurements for the new drug correct systematic compound-level bias (18–20). This setting corresponds closely to experimental planning, because a limited screening campaign can be followed by model-assisted mapping of the remaining process domain.

Hyperparameter selection also affects the apparent ranking of learning algorithms. Bayesian and population-based optimizers differ in sampling efficiency, global exploration, and sensitivity to mixed search spaces (21–25). Direct comparison under common folds and evaluation budgets is therefore needed before attributing superiority to either a model or an optimizer. XGBoost and CatBoost offer regularized boosting strategies with different treatments of residual structure, while multilayer perceptrons provide a flexible smooth nonlinear alternative (26–29).

This study addresses that gap through a leakage-controlled benchmark crossing XGBoost, CatBoost, and multilayer perceptron regression with tree-structured Parzen estimation (TPE), differential evolution (DE), and particle swarm optimization (PSO). The methodological contributions are fourfold: (i) complete-drug grouped validation prevents observations from the same compound from appearing in both model development and outer testing; (ii) nested grouped cross-validation confines hyperparameter selection to the outer-training drugs; (iii) a few-shot protocol quantifies the incremental value of 0, 1, 3, and 5 drug-specific calibration measurements without retraining the global model; and (iv) TPE, DE, and PSO are compared under identical folds, seeds, search spaces, and computational budgets. Log-scale learning addresses the 0.0002–15.7 g/L response range, while drug-macro and robust relative-error measures complement conventional metrics. The resulting design separates interpolation from chemical transfer and provides a reproducible basis for deciding how much new experimental information is required before machine-learning predictions become practically informative.

## 2 Materials and methods

### 2.1 Dataset and provenance

The dataset was obtained from the workbook reported by Bahrami et al. (1). The data available to the present study contains exactly 1,618 rows representing 60 drugs. Although the associated publication describes a larger compiled collection, no additional records were present in the supplied workbook; therefore, the analysis used every available row in that file, with no extra inclusion, exclusion, or subsampling criteria imposed by the present study. Each row includes drug name, molecular weight (MW), melting point (MP), temperature (T), pressure (P), and solubility (S) in g/L. MW and MP were used as the only drug-specific numerical descriptors because they were the molecular/solid-state descriptors consistently available for all 60 drugs in the supplied workbook. Additional properties such as polarity, crystal structure, hydrogen-bonding descriptors, fingerprints, and other solid-state variables were not consistently available and were not imputed from external sources, thereby avoiding a second, potentially heterogeneous data-curation layer. Drug name was used only to define groups and calibration tasks, not as a predictive input. The numerical ranges are summarized in Table 1, and the broad target distribution is shown in Figure 1.

**Table 1:** Descriptive ranges of the analyzed dataset.

Variable	Minimum	Maximum	Mean	Median
MW (g/mol)	166.2	853.9	417.6	419.9
MP (K)	293	589	464.5	472.8
Temperature (K)	308	348.2	324.2	328
Pressure (bar)	100	400	209.5	210
Solubility (g/L)	0.0002	15.7	0.7576	0.15

**Figure 1:**
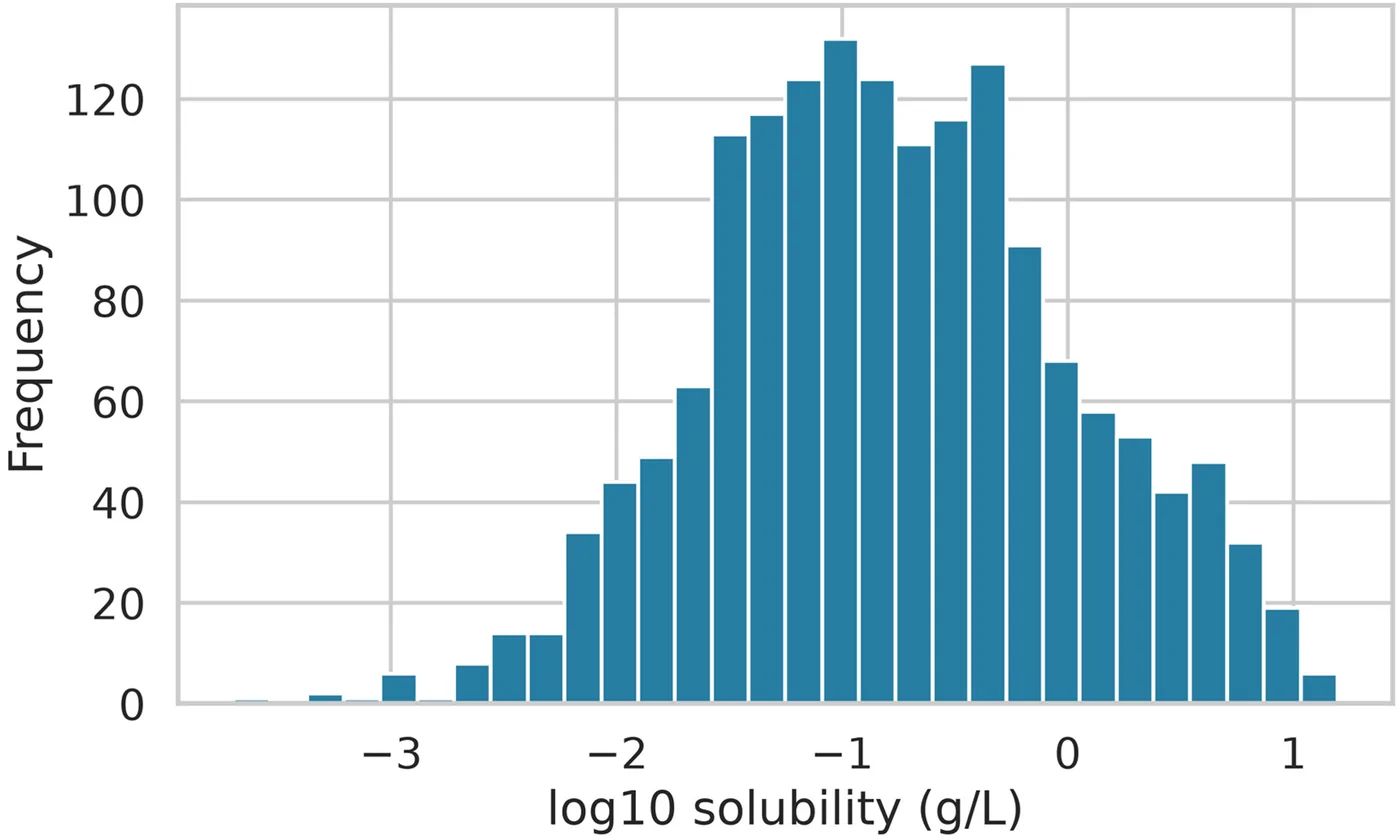
Distribution of log₁₀-transformed drug solubility across the 1,618 observations (1).

### 2.2 Data integrity and response transformation

Column names, data types, missingness, positivity of S, row count, and the number of unique drugs were checked before modeling. No missing values were detected, and no observation was automatically removed. Because S spans approximately 78,500-fold, direct minimization of squared error in g/L would overemphasize the largest responses. The target was therefore transformed according to Equation (1): On the original scale, a small number of highly soluble observations can contribute disproportionately to squared-error objectives and obscure relative performance among low-solubility drugs. The log₁₀ transformation compresses this range and converts multiplicative deviations (for example, predicting twofold too high or too low) into more symmetric additive errors, producing a more balanced learning problem across the solubility spectrum.

$$z=log_{10}(S),\;S>0$$ (1)

Predictions were returned to the original unit using Ŝ = 10^ẑ. A residual-smearing correction can address retransformation bias (30), but it was not applied because the prespecified selection objective was defined on the log scale and no correction was tuned using held-out drugs. Feature scaling was fitted within each training partition for the multilayer perceptron only. Tree models used unscaled numerical inputs. All preprocessing, optimization, and model fitting were completed independently inside each outer training fold. In particular, estimating or selecting a retransformation correction using information from an outer held-out drug would introduce test-drug information into model development and weaken the strict leakage-controlled validation design; for this reason, no held-out-drug-dependent smearing adjustment was introduced.

### 2.3 Nested grouped validation and few-shot protocol

Five-fold GroupKFold cross-validation was used for the outer evaluation; each drug appeared in exactly one outer test fold. Hyperparameters were selected through three-fold grouped cross-validation using only the outer-training drugs. The same outer folds, inner folds, random seeds, and optimizer budgets were used for all model–optimizer combinations (Table 2). After zero-shot prediction of each held-out drug, k = 1, 3, or 5 observations were sampled as calibration measurements and excluded from evaluation. Sampling was repeated ten times at each k. A shrinkage correction was estimated on the log scale using Equations (2) and (3): In plain terms, the few-shot step uses the observed calibration points from a held-out drug to estimate only whether that drug tends to sit systematically above or below the global log-solubility surface. This drug-specific shift is then applied to the remaining observations of that drug while the globally learned pressure-temperature response and all model parameters remain unchanged.

$$\delta_{k}\;=\;[k/(k\;+\;\lambda )]\;\cdot \;(1/k)\;\Sigma_{i=1}^{k}\;(z_{i}\;-{\hat{z}}_{i})$$ (2)

$$\hat{S}adapted=10\;\hat{\;}\;(\hat{z}+\;\delta_{k})$$ (3)

**Table 2:** Prespecified model-development and evaluation design.

Component	Specification
Predictors	MW, MP, temperature, and pressure
Target	log₁₀(S), inverse-transformed for reporting
Outer validation	Five grouped folds; complete drugs held out
Inner validation	Three grouped folds within each outer-training set
Models	XGBoost, CatBoost, and multilayer perceptron
Optimizers	TPE, DE, and PSO
Optimization budget	10 objective evaluations per model–optimizer–outer-fold task
Calibration	0, 1, 3, and 5 observations; 10 repeated draws for k > 0
Primary selection metric	Drug-macro log-MAE plus 0.1 × fold-to-fold SD

A fixed *λ* = 2 was prespecified as a conservative shrinkage strength to prevent a single calibration observation, or a very small calibration set, from producing an excessive drug-specific correction. Keeping *λ* fixed also avoids tuning the calibration strength with information from the outer test drug, which would compromise the intended separation between model development and held-out evaluation. At k = 0, *δ*₀ = 0. This mechanism changes only the drug-specific intercept on the log scale; it does not retrain model parameters or use the evaluation observations.

### 2.4 Machine-learning models

#### 2.4.1 XGBoost

XGBoost constructs an additive ensemble of regression trees and regularizes tree complexity (26). At boosting step t, a new tree fₜ is added to reduce the loss while penalizing the number and magnitude of terminal-node weights (Equation (4)):

$$L(t)\;=\;\Sigma_{i}\;l(z_{i},{\hat{z}}_{i}(t-1)\;+\;f_{t}(x_{i}))\;+\;\gamma T\;+\;(\eta /2)\Sigma_{j}w_{j}^{2}$$ (4)

For XGBoost, the optimized hyperparameters were the number of trees, maximum depth, learning rate, row subsampling, feature subsampling, minimum child weight, and L1/L2 regularization. The model used a pseudo-Huber objective to reduce sensitivity to large log-scale residuals. Overfitting was controlled through depth and child-weight constraints, row/feature subsampling, explicit L1/L2 regularization, and nested grouped validation for hyperparameter selection.

#### 2.4.2 CatBoost

CatBoost is a gradient-boosting implementation that uses symmetric trees and ordered boosting strategies (27). Although no categorical predictor was supplied to the model, its regularized tree construction provides a useful contrast with XGBoost. The additive prediction is expressed in Equation (5):

$$\hat{z}(x)\;=\;\Sigma_{t=1}^{M}\alpha \;f_{t}(x)$$ (5)

For CatBoost, the optimized hyperparameters were the number of iterations, tree depth, learning rate, L2 leaf regularization, random strength, and bagging temperature. Huber loss was used so that small residuals retain approximately quadratic behavior while larger residuals receive more robust, approximately linear treatment (31). Overfitting was controlled through symmetric-tree depth, L2 leaf regularization, stochasticity induced by random strength and bagging temperature, and nested grouped validation.

#### 2.4.3 Multilayer perceptron

The multilayer perceptron (MLP) represents the response through stacked affine transformations and nonlinear activations (28, 29). For layer ℓ, the hidden representation is given by Equation (6):

h(ℓ)=φ(W(ℓ)h(ℓ−1)+b(ℓ)) (6)

For the MLP, candidate architectures contained one to three hidden layers, and the optimized hyperparameters were hidden-layer width, activation function, L2 penalty, initial learning rate, and batch size. The network was trained as a continuous regression model using squared-error loss on the transformed target. Standardization parameters were learned exclusively from the current training fold. L2 regularization, early stopping, and fold-specific preprocessing were the principal overfitting controls, with all architecture and training choices selected only inside the nested grouped-validation procedure.

### 2.5 Hyperparameter optimization

TPE models favorable and unfavorable regions of the search space and proposes configurations that increase their density ratio (21, 22). DE evolves a population using scaled vector differences and crossover (23), whereas PSO updates particles using inertial, personal-best, and global-best components (24). Each optimizer received ten objective evaluations for every model and outer fold. This deliberately constrained and identical budget was chosen to keep the full nested benchmark computationally tractable and, more importantly, to ensure that optimizer comparisons reflected sample efficiency under equal opportunity rather than unequal search effort. The budget was not intended to identify each optimizer's asymptotic optimum. The objective combined mean drug-level absolute error on z with a stability term (Equation (7)):

J=mean_d(MAElog,d)+0.1⋅SD_folds(MAElog,d) (7)

Drug-macro log-MAE was used because each held-out drug should contribute equally to model selection, regardless of how many pressure-temperature observations it contains; a pooled row-level error would give disproportionate influence to drugs represented by more rows. The 0.1 × fold-to-fold SD term provides a modest stability penalty so that configurations with similar mean error are favored when their performance is more consistent across grouped validation folds. The complete computational sequence is illustrated in Figure 2. Optimization histories, chosen parameters, group assignments, and row-level predictions were written directly by the analysis script.

**Figure 2:**
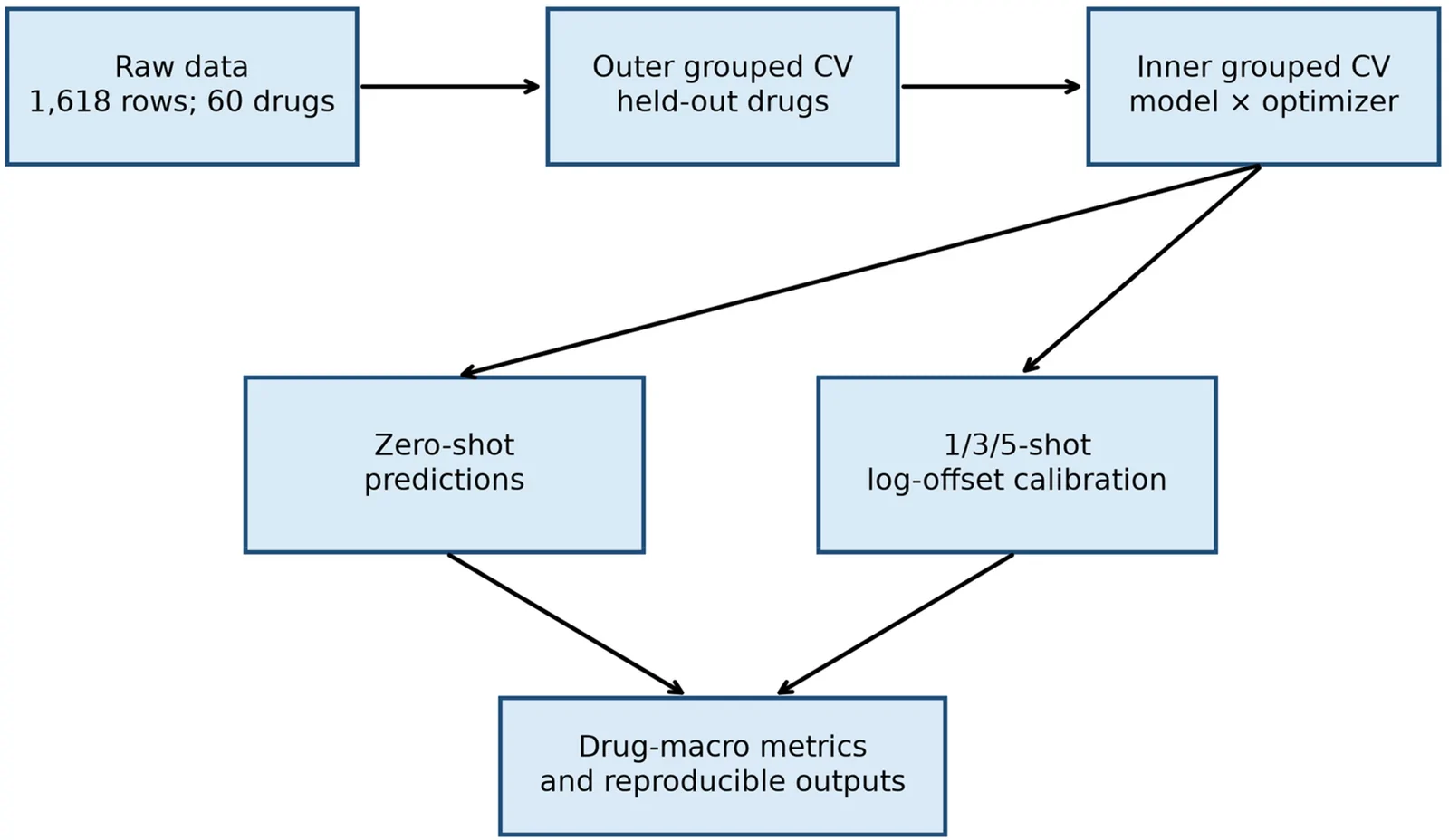
Leakage-controlled nested validation and few-shot calibration workflow.

### 2.6 Performance measures

Model performance was summarized using R², RMSE, MAE, average absolute relative deviation (AARD%), median absolute relative deviation (MdARD%), log-RMSE, log-MAE, drug-macro MAE, and worst-drug MAE, following standard regression-evaluation principles (32). These metrics were interpreted as complementary rather than interchangeable: R² describes explained variance relative to the observed response, RMSE places greater weight on large absolute errors in g/L, MAE summarizes typical absolute deviation in the original unit, and log-MAE/log-RMSE characterize multiplicative error after accounting for the wide response range. Drug-macro MAE gives each compound equal influence, while worst-drug MAE exposes difficult transfer cases. AARD% can become extremely large when S is close to zero because of the small denominator, so MdARD% provides a more robust summary of relative error and was interpreted together with the log-scale metrics. The principal equations for RMSE and AARD were Equations (8) and (9), respectively:

$$RMSE\;=\;\surd [(1/n)\Sigma_{i}{\left(y_{i}\;-{\hat{y}}_{i}\right)}^{2}]$$ (8)

$$AARD(\text{\%})\;=\;(100/n)\Sigma_{i}\;|(y_{i}\;-\;{\hat{y}}_{i})/y_{i}|$$ (9)

## 3 Results

### 3.1 Zero-shot transfer to unseen drugs

The zero-shot results are presented in Table 3 and visualized as a model–optimizer heatmap in Figure 3. Under the present dataset, grouped folds, primary metric, and ten-evaluation tuning budget, XGBoost–TPE achieved the lowest log-MAE (0.584), followed by CatBoost–PSO (0.590) and MLP–TPE (0.598). Nevertheless, all zero-shot R² values were near or below zero. The lowest-log-MAE configuration yielded R² = −0.087, RMSE = 1.713 g/L, and MdARD = 82.4%. The near-zero or negative R² values are scientifically informative: even flexible nonlinear learners and optimizer tuning could not recover compound-specific solubility from MW, MP, temperature, and pressure when the target drug was entirely absent from model development. This indicates that the principal limitation is missing molecular and solid-state information rather than insufficient nonlinear model capacity. Thus, optimizer selection produced modest differences within this benchmark, but none of the investigated algorithms provided reliable absolute zero-shot transfer to a wholly unseen drug from the available descriptors.

**Table 3:** Zero-shot performance for complete-drug holdout evaluation.

Model	Optimizer	R²	RMSE	MAE	MdARD (%)	log-MAE
XGBoost	TPE	−0.087	1.713	0.721	82.4	0.584
CatBoost	PSO	−0.015	1.655	0.675	88.2	0.590
MLP	TPE	0.017	1.629	0.691	89.5	0.598
CatBoost	DE	−0.031	1.669	0.686	91.3	0.620
CatBoost	TPE	−0.050	1.684	0.690	91.1	0.620
XGBoost	PSO	−0.100	1.723	0.732	88.3	0.622
MLP	PSO	−3.007	3.289	1.071	93.8	0.642
XGBoost	DE	−0.855	2.238	0.934	82.5	0.648
MLP	DE	−0.250	1.837	0.800	94.0	0.650

**Figure 3:**
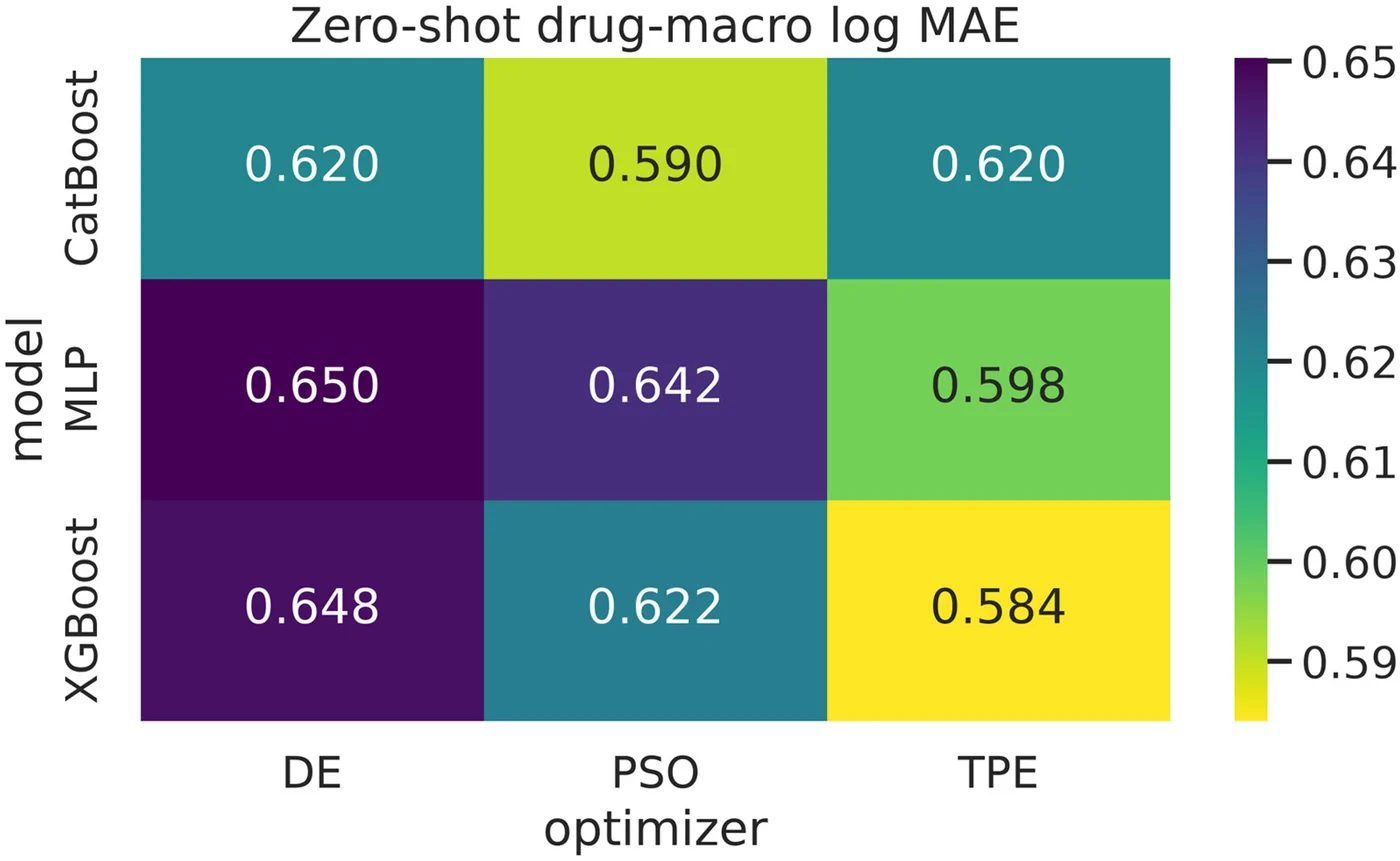
Zero-shot drug-macro log-MAE across the three models and three hyperparameter optimizers.

### 3.2 Effect of sparse drug-specific calibration

Sparse calibration consistently reduced log-scale error (Table 4; Figure 4). Under the present evaluation framework, CatBoost–PSO produced the lowest one-shot log-MAE (0.418) and improved R² to 0.166, whereas XGBoost–TPE produced the lowest log-MAE at three and five observations. At five shots, its log-MAE was 0.251, representing a 57.0% reduction relative to the zero-shot value, while RMSE decreased by 28.6% and MdARD fell from 82.4% to 42.9%. The improvement was largest when moving from zero to the first few calibration measurements and became progressively smaller thereafter, indicating that even a very small experimental campaign supplies substantial compound-specific information while additional measurements provide diminishing incremental gains. These gains arose from correction of compound-level bias rather than access to the remaining test responses.

**Table 4:** Lowest-error configuration under the present protocol at each calibration level.

Shots	Model	Optimizer	R²	RMSE	MAE	MdARD (%)	log-MAE
0	XGBoost	TPE	−0.087	1.713	0.721	82.4	0.584
1	CatBoost	PSO	0.166	1.496	0.563	71.6	0.418
3	XGBoost	TPE	0.317	1.371	0.485	50.3	0.298
5	XGBoost	TPE	0.452	1.223	0.430	42.9	0.251

**Figure 4:**
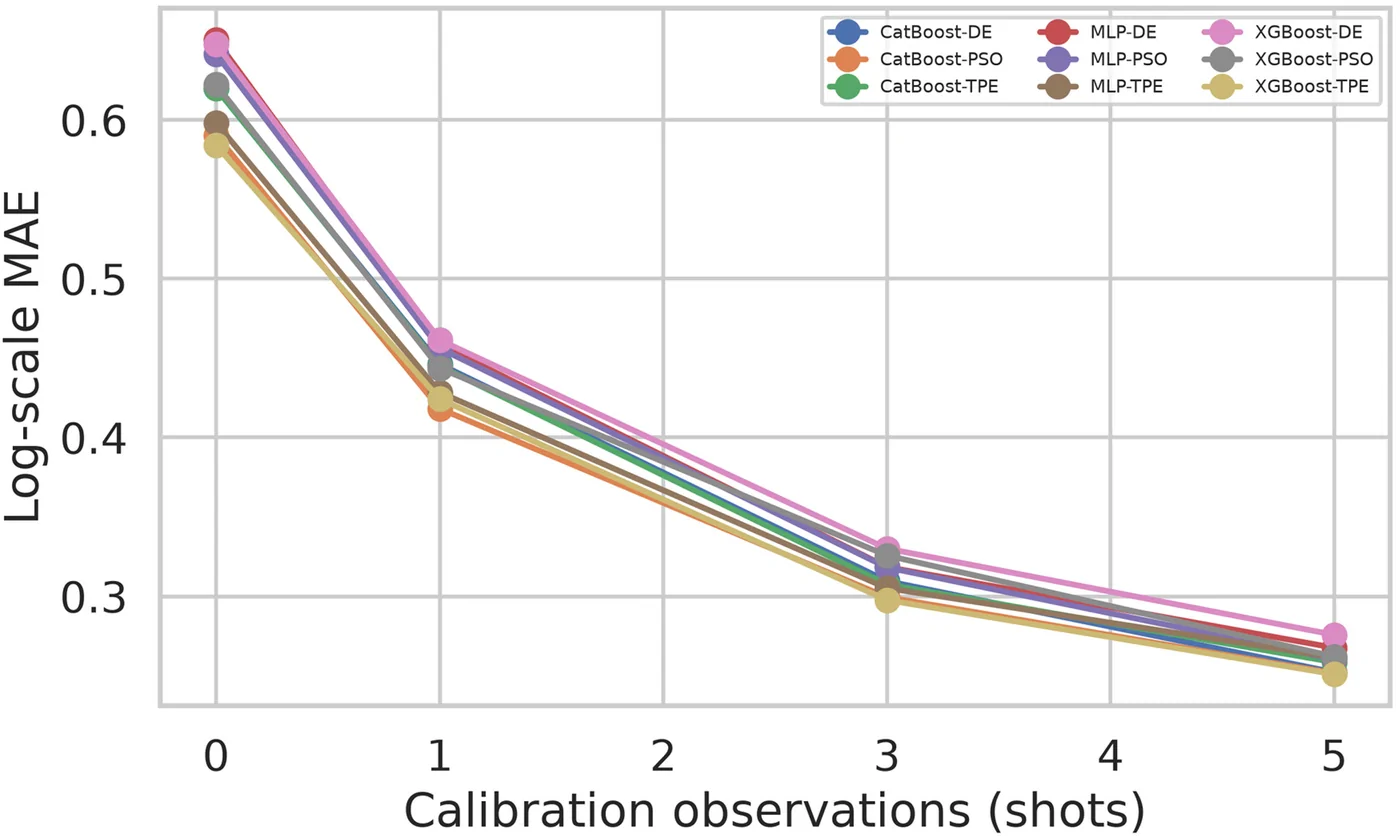
Change in log-MAE as the number of drug-specific calibration measurements increases.

### 3.3 Optimizer behavior and selected hyperparameters

Figure 5 shows the lowest inner-validation objective attained as evaluations accumulated. Within the declared dataset, grouped folds, search spaces, metric, and ten-evaluation budget, TPE identified the lowest-objective XGBoost settings at the calibration levels where that model attained the lowest reported error, whereas PSO identified the lowest-error one-shot CatBoost configuration. The limited budget was intentionally equal across methods, so the comparison reflects sample efficiency under constrained tuning rather than asymptotic optimizer performance. Table 5 reports the fold-specific XGBoost–TPE settings, illustrating that no single configuration dominated every chemical partition.

**Figure 5:**
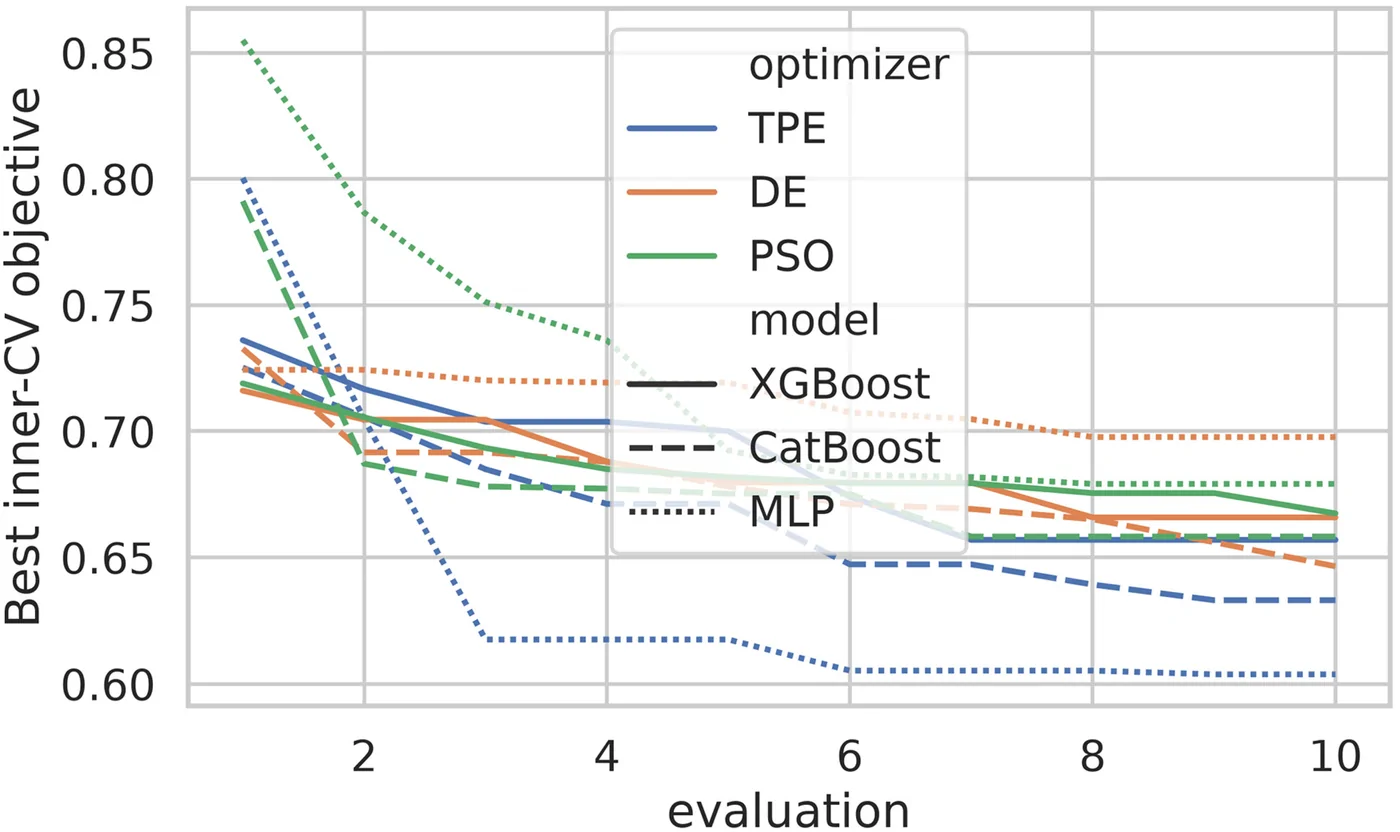
Lowest inner-cross-validation objective as a function of optimizer evaluations.

**Table 5:** Fold-specific hyperparameters selected for XGBoost–TPE.

Fold	Trees	Depth	Learning rate	Subsample	Feature fraction	L2
0	167	2	0.0303	0.675	0.980	6.4
1	320	3	0.0109	0.910	0.734	0.00533
2	302	4	0.0109	0.972	0.790	41.8
3	124	7	0.0260	0.984	0.692	45.4
4	377	7	0.0211	0.670	0.675	0.00161

### 3.4 Prediction patterns for the lowest-error five-shot configuration under the present protocol

The parity plot for the five-shot configuration with the lowest reported log-MAE under the present protocol is shown in Figure 6. Predictions follow the overall order of magnitude across the response range, but errors remain larger for the highest-solubility compounds. This behavior is also reflected by the worst-drug MAE of 4.255 g/L. The log transformation prevents high-solubility observations from dominating training, but it cannot supply chemical information absent from MW and MP. Therefore, improvement in central and relative accuracy does not imply uniformly reliable predictions for every drug.

**Figure 6:**
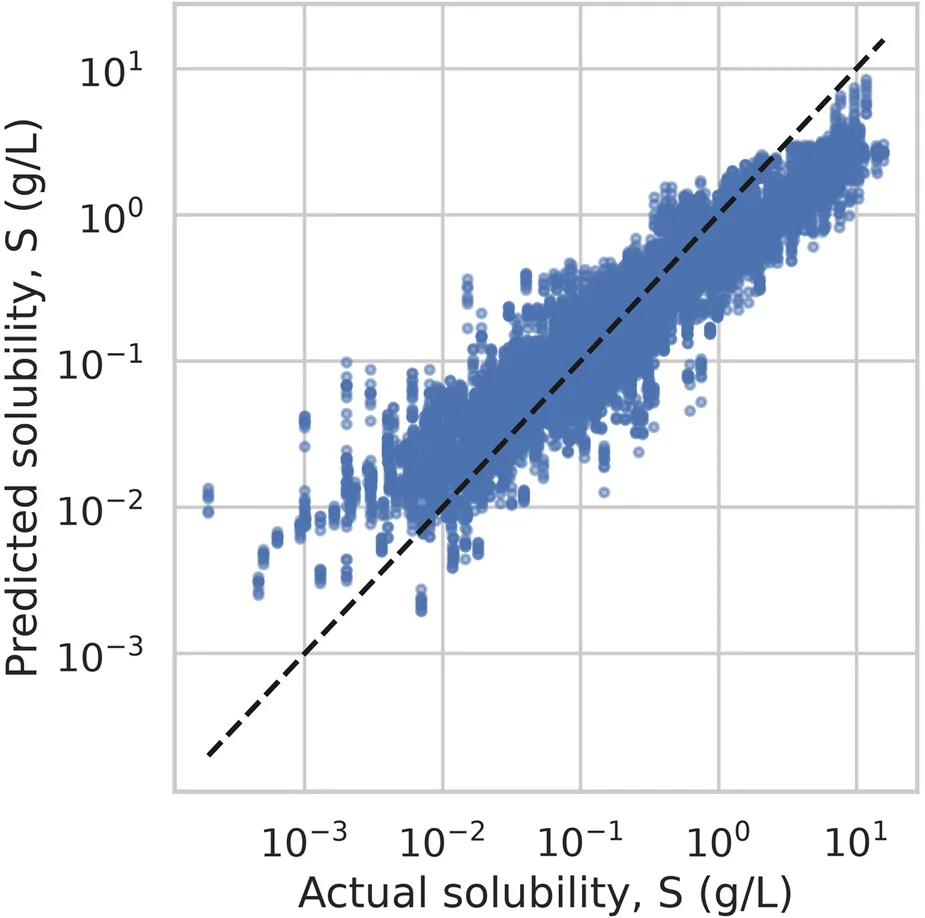
Actual versus predicted solubility for the lowest-error five-shot configuration under the present protocol. Both axes are logarithmic.

## 4 Discussion

The principal result is that sparse calibration changes the practical interpretation of multidrug solubility modeling. Without any measurements for the target drug, the investigated descriptors did not support dependable transfer, despite flexible nonlinear learners and optimizer tuning. The near-zero or negative zero-shot R² values should therefore be interpreted as a scientific result rather than merely as an unsuccessful prediction scenario: they show that model flexibility cannot compensate for molecular and solid-state information that is absent from the predictor set. This outcome is consistent with the chemical diversity of pharmaceutical solids: equal or similar MW and MP values do not uniquely encode crystal packing, polarity, hydrogen bonding, sublimation pressure, or specific interactions with CO_2_ (2–6). Random-row validation can conceal this limitation because neighboring pressure–temperature observations from the same drug remain available during training (14–17).

One to five measurements provide direct information about the target compound's solubility scale. The proposed shrinkage offset uses that information conservatively and preserves the global pressure–temperature response learned from other drugs. The largest practical gain occurs with the first few measurements, after which the reductions in log-MAE become progressively smaller; this pattern suggests that sparse observations rapidly identify much of the compound-level displacement on the log scale, while later measurements mainly refine that estimate. Importantly, this strategy differs from conventional drug-specific model retraining: it does not refit trees, neural-network weights, or pressure-temperature relationships for the new compound. Instead, it adjusts only a single compound-specific log-scale offset, so it can operate with substantially less target-drug information than would be required to fit a new model from scratch. Experimentally, a small calibration design spanning the intended conditions can therefore anchor the global model before it is used to interpolate a denser operating map. Future work should optimize the selection of calibration conditions rather than sample them randomly.

The optimizer comparison also cautions against presenting a model name as sufficient methodological detail. Under the present dataset, grouped folds, calibration protocol, metric, and ten-evaluation budget, XGBoost–TPE attained the lowest reported error at zero, three, and five shots, whereas CatBoost–PSO attained the lowest one-shot error. With only ten evaluations, the algorithms differed in how efficiently they navigated the search spaces. TPE can concentrate on promising conditional regions (21, 22), whereas DE and PSO maintain population diversity (23, 24). These results do not establish universal optimizer superiority; they identify the lowest-error combinations under the declared computational budget and folds.

AARD% was extremely large in the zero-shot setting because observations near 0.0002 g/L create very small denominators. MdARD and log-MAE are consequently more stable indicators for this dataset. Reporting only R² would also be incomplete: R² improved with calibration but remained moderate, whereas log-MAE showed a clear reduction in multiplicative error. The combined metric set exposes both the benefit and the remaining uncertainty.

Compared with equation-of-state and semi-empirical correlations, the present method does not require compound-specific critical-property estimates or vapor-pressure parameters (3–6). Conversely, thermodynamic models encode physical structure and may extrapolate more plausibly when their parameters are reliable. Machine learning should therefore be viewed as complementary: it can rapidly interpolate after sparse calibration, while thermodynamic analysis remains valuable for mechanistic interpretation and extrapolation. A direct numerical claim of superiority over published thermodynamic models was avoided because different studies use different compounds, units, data partitions, and calibration information.

Several limitations define the scope of the present conclusions and priorities for future work. First, descriptor availability was restricted to MW, MP, temperature, and pressure; richer molecular descriptors, fingerprints, polarity and hydrogen-bonding measures, solid-state properties, and CO_2_ density may improve chemical transfer. Second, the dataset is literature-derived and may contain interlaboratory heterogeneity in measurement protocols and reporting. Third, evaluation was internal to the 60 drugs in the supplied workbook and therefore does not constitute prospective external validation. Fourth, calibration observations were sampled randomly rather than selected through an optimized experimental-design strategy. Fifth, some MLP candidates reached the maximum iteration count, although early stopping and nested evaluation limited the effect of possible convergence or overfitting problems. These limitations do not invalidate the leakage-controlled comparison performed here; rather, they specify the conditions under which the results should be interpreted and motivate prospective validation, richer descriptors, optimized calibration-point selection, and further investigation of neural-network convergence.

## 5 Conclusion

A reproducible nested group-validation study compared XGBoost, CatBoost, and MLP regression tuned by TPE, DE, and PSO for multidrug solubility in scCO_2_. Modeling log₁₀(S) accommodated the 0.0002–15.7 g/L range without deleting valid extremes. Zero-shot transfer to unseen drugs was poor, demonstrating that molecular weight, melting point, temperature, and pressure alone do not identify compound-specific solubility. Sparse calibration changed this result: under the present grouped protocol, five measurements reduced the lowest observed log-MAE from 0.584 to 0.251 and increased the corresponding R² from −0.087 to 0.452. The appropriate conclusion is therefore not that a global model replaces experiments, but that a small, strategically designed measurement set can anchor machine-learning interpolation for a new drug. Prospective validation with richer molecular and thermodynamic descriptors is required before process-design use.

## Data Availability

The original contributions presented in the study are included in the article/Supplementary Material, further inquiries can be directed to the corresponding author.
